# Detailed chemical analysis of a fully formulated oil using dielectric barrier discharge ionisation–mass spectrometry

**DOI:** 10.1002/rcm.9320

**Published:** 2022-05-16

**Authors:** Vincent Basham, Tom Hancock, John McKendrick, Nathalia Tessarolo, Chrissie Wicking

**Affiliations:** ^1^ Department of Chemistry, School of Chemistry, Food and Pharmacy, Whiteknights Campus University of Reading Reading UK; ^2^ BP Technology centre Pangbourne UK

## Abstract

**Rationale:**

Fully formulated oils (FFOs) are integral to automotive lubrication; however, detailed compositional analysis is challenging due to high levels of chemical complexity. In particular, existing mass spectrometric approaches often target particular FFO components, leading to poor analytical coverage of the overall formulation, with increased overheads and analytical timescales.

**Methods:**

Herein we report the application of a commercially available SICRIT SC‐20 dielectric barrier discharge ionisation (DBDI) source and Thermo Fisher Scientific LTQ Orbitrap XL to the analysis of an FFO. Nitrogen was used as a discharge gas for the DBDI source, and was modified using a range of commonplace solvents to tailor the experimental conditions for the analysis of various components.

**Results:**

The reported method allowed analysis of a range of FFO components of interest, encompassing a wide range of chemistries, in under 1 min. By modifying the discharge gas used for ionisation, experiments could be optimised for the analysis of particular FFO components across positive and negative ion modes. In particular, use of water vapour as a discharge gas modifier with positive ion mode mass spectrometry permitted concomitant analysis of antioxidants and base oil hydrocarbons. Furthermore, case studies of selected linear alkanes and alkenes profile the differences in the range of ions formed across these saturated and unsaturated aliphatic compounds, giving insight into the fate of base oil hydrocarbons in FFO analyses.

**Conclusions:**

A rapid method for analysis of FFO compositions has been developed and provides coverage of a range of components of interest. The results indicate that the method presented may be of utility in analysis of other FFOs or similarly challenging complex mixtures.

## 1 INTRODUCTION

Engines are mechanical environments that need lubrication in order to maximise efficiency, reduce friction, suspend particulates and extend component lifetimes. This requirement is fulfilled by the use of fully formulated oils (FFOs), which are petrochemical products comprised of base oil and a range of carefully selected additives. Base oil is the major component of an FFO and is a semi‐viscous range of hydrocarbons of either mineral or synthetic origin. A balanced range of speciality chemicals are admixed into this base oil as additives, which tailor the formulation to a particular application by altering the physical and chemical properties of the oil. Common additives include antioxidants, detergents, dispersants, antifoaming agents, viscosity modifiers and zinc dialkyldithiophosphates (ZDDPs), amongst others.^1^, ^2^, ^3^, ^4^

Owing to the overall complexity of the resultant product, obtaining detailed information on the chemical composition of FFOs using a single analytical technique is particularly challenging. Analysis of highly complex mixtures is a strength of mass spectrometry (MS), particularly when using mass analysers of high resolving power, or when coupled to a chromatographic technique such as gas or liquid chromatography. A range of ion sources are available to tailor an instrument to a particular application. Of those commonly available, electrospray ionisation (ESI) has well‐documented use in the analysis of particular additives within FFOs.^5^, ^6^

Reported in the literature is the application of dielectric barrier discharge ionisation (DBDI)‐MS to a range of analytes not easily accessible by ESI‐MS, such as fluorinated alkanes and polycyclic aromatic hydrocarbons.^7^, ^8^ Dielectric barrier discharge sources are relatively simple and operate via the generation of a low‐temperature plasma. Briefly, two electrodes separated by a dielectric barrier are held at high voltage, and the ionisation of the proximal discharge gas leads to the generation of charged species such as N_2_^•+^, O_2_^•+^ and NO_2_^−^, amongst others.^7^, ^9^, ^10^ Of these, N_2_^•+^ is principally responsible for much of the subsequent ionisation of analytes through reactions such as proton transfer, charge transfer, electron capture, hydride transfer and ion attachment.^11^ DBDI sources are often set up to allow ambient sampling via the direction of the plasma at samples deposited on surfaces. In such a manner, the analysis of various engine oils available on the market has been reported, whereby the authors used a simple experimental set‐up to allow chemometric fingerprinting of samples.^12^

An alternative DBDI source design has been reported which instead uses an in‐line geometry to minimise ion loss due to repulsion. Not only does the development of a non‐ambient DBDI source allow coupling to more automated sampling methods, such as gas chromatography (GC) and solid‐phase microextraction (SPME), but also allows low limits of detection for particular analytes to be demonstrated.^13^, ^14^ A range of alkanes, including those in a diesel sample, have been effectively quantified using GC coupled to this source design.^15^

Herein the application of DBDI‐MS in determining the chemical composition of an FFO is reported. A commercially available experimental set‐up has been adapted to allow analysis of an FFO using active capillary DBDI in both positive and negative ion mode MS, directly from appropriately diluted liquid samples. We show that this method is advantageous over more commonplace MS techniques such as ESI or field ionisation (FI) for analysing selected FFO components within a single run.

## 2 EXPERIMENTAL

### 2.1 Chemicals and samples

Water, methanol and acetonitrile were purchased from Fisher Chemicals (Loughborough, UK) with LC/MS‐grade purity. Isopropanol and toluene were purchased from Honeywell Riedel‐de Haën (Loughborough, UK), also with LC/MS‐grade purity. FFO components were supplied by BP Castrol (Pangbourne, UK) and prepared in‐house to resemble an FFO according to the formulation reported below. *n*‐Decane, 1‐decene, *n*‐dodecane and 1‐dodecene were all purchased from Fisher Chemicals (Loughborough, UK) and diluted 1 in 10 using toluene for analysis. *n*‐Decane and *n*‐dodecane were of 99% purity, whilst 1‐decene and 1‐dodecene were of 96% purity (remainder isomers).

### 2.2 Formulation

For each additive, between 110 and 123 mg of material was made up to 1 mL using toluene and vortexed to complete dissolution. From this, 1 mL of an FFO was prepared according to the formulation described in Table 1. For analysis, 125 μL of this solution was made up to 1 mL using toluene.

**TABLE 1:** Formulation of FFO

Code	Chemical ID	Amount (vol%)
001	Base oil (Group III)	80–85
002	Antifoam	0.001–0.005
003	Phenolic antioxidant	0.1–1.0
004	Aminic antioxidant	0.1–1.0
005	Dispersant	5–10
006	Sulphonate detergent 1	0.1–1.0
007	Sulphonate detergent 2	0.1–1.0
008	Phenate detergent	0.1–1.0
009	Viscosity modifier	5–10
010	Secondary ZDDP	0.1–1.0

Formulation used to prepare 1 mL of dilute FFO.

### 2.3 Mass spectrometry

All MS analyses were conducted using a Thermo Fisher Scientific LTQ Orbitrap XL with a scan range of 80–2000 *m*/*z*, a resolution of 30 000 and capillary temperature of 275°C. A maximum ion injection time of 500 ms and automatic gain control target of 10^6^ were used in all instances. The auxiliary gas flow was set to 5 arbitrary units in all instances and used to supply the DBDI source with a controlled N_2_ gas flow. The tube lens was set to 110 V for positive ion mode analyses and −90 V for negative ion mode analyses. Spectra were recorded for 30 scans in the instance of blank analyses, or continuously for 1 min for FFO samples.

A commercially available SICRIT SC‐20 and complementary SPME heating unit (Plasmion GmbH, Augsburg, Germany) were used for all analyses. Within the SPME heating unit, a Topaz 4.0 mm ID Precision Inlet Liner w/Wool (Restek Ltd, Buckinghamshire, UK) was used to ensure rapid evaporation of sample material. The auxiliary gas from the MS interface was either routed directly into the source in the instance of a pure N_2_ discharge gas, or through a 1 L bubbler filled with approximately 300 mL of the corresponding solvent in the instance of modified discharge gases. A depiction of the set‐up is included in Figure S1. The source voltage was set at 1.5 kV for positive ion mode analyses and 1.7 kV for negative ion mode analyses. The SPME heating unit was held at 275°C for all analyses, unless otherwise specified, and further modified by the attachment of a Varian CP3800 GC injector septum nut containing a 9 mm non‐stick BTO septum (Restek Ltd, Buckinghamshire, UK) to ensure airtight sample injection. Analyses were conducted by injection of 1 μL of diluted FFO sample into the SPME heating unit from a gastight GC syringe.

### 2.4 Data visualisation

Initial data analysis was conducted in the native Qual Browser (version 3.0.63) MS software package (Thermo). Manipulation and graphical visualisation of the datasets were performed in Python (version 3.8.3) using the seaborn (version 0.10.1), matplotlib (version 3.2.2) and pandas (version 1.0.5) libraries. All Python scripts for handling of exported data and preparing Kendrick mass defect (KMD), van Krevelen and composition bar plots were written in‐house.

## 3 RESULTS AND DISCUSSION

### 3.1 Positive ion mode DBDI‐MS

Antioxidants and base oil within formulations were successfully identified using DBDI‐MS in positive ion mode. Initial investigations comparing the use of both dry N_2_ and N_2_ humidified with water vapour as a discharge gas yielded [M + H]^+^ ions for aminic antioxidants. The protonation of amine‐containing analytes both with and without water vapour present in an active capillary set‐up has been documented, with evidence suggesting that, unlike atmospheric pressure chemical ionisation, the formation of hydroxonium clusters is not a requisite of this ionisation pathway.^16^

**FIGURE 1:**
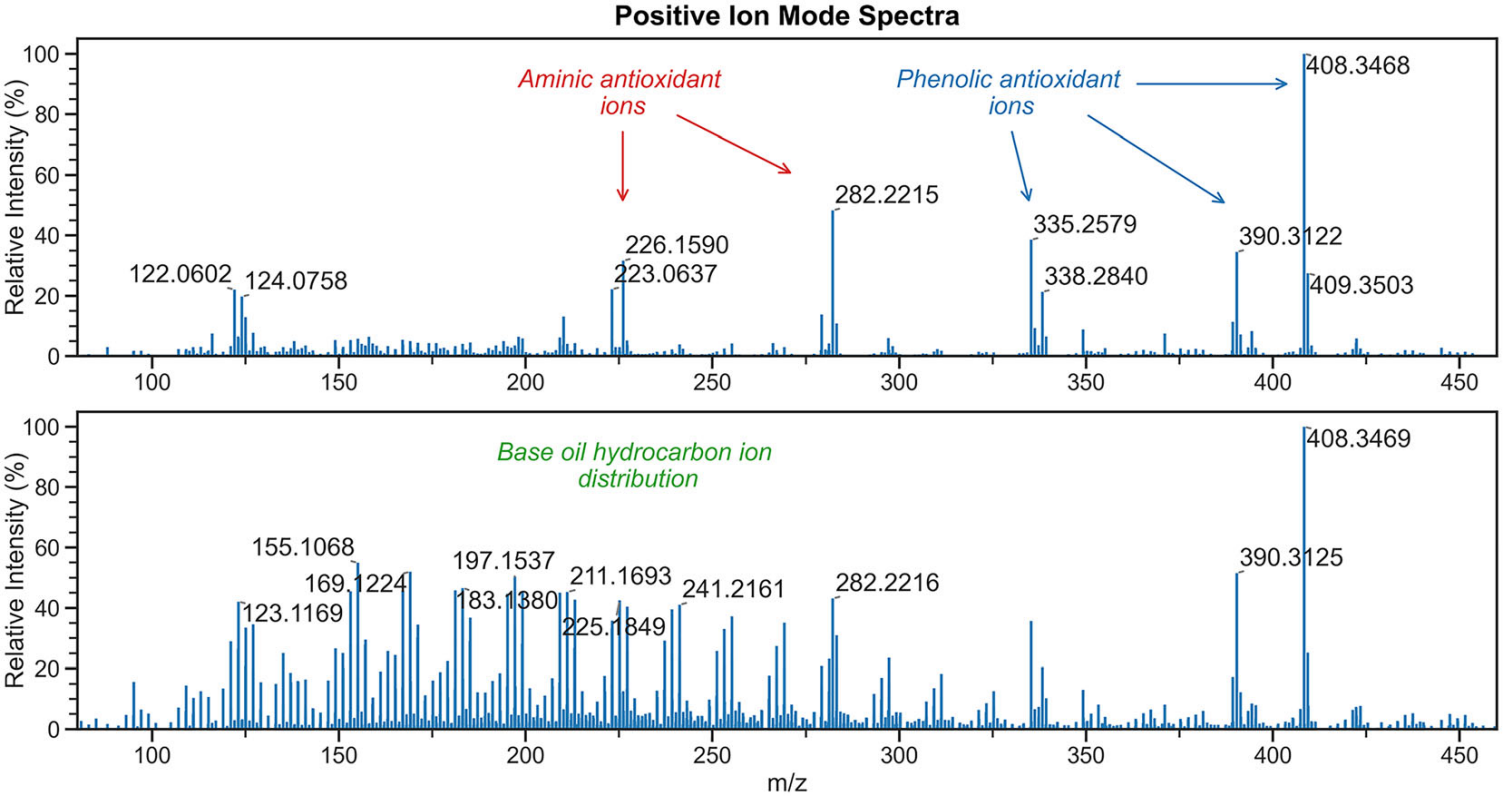
Mass spectra of FFO generated in positive ion mode. Range of antioxidant ions observed at 0.10 min when analysing an FFO (top) and distribution of hydrocarbon‐based ions observed at 0.40 min in the same analysis (bottom), when using N_2_ humidified with water vapour as a discharge gas. Residual antioxidant ions can be observed in the bottom spectrum. Delayed ionisation of hydrocarbon base stock is attributed to eventual vaporisation within the SPME unit upon continued heating [Color figure can be viewed at wileyonlinelibrary.com]

Unlike the formation of simple protonated ions for aminic antioxidants under both humidified and non‐humidified conditions, the ions generated for the phenolic antioxidant are dependent on water vapour, or lack thereof, within the discharge gas. Without humidification of the discharge gas, [M − CH_3_]^+^ ions are observed for the phenolic antioxidant, whereas the use of a humidified gas yields [M]^•+^ and [M − C_4_H_9_]^+^ ions, in addition to [M + NH_4_]^+^ adducts. When the source is supplied with dry N_2_, the exclusive formation of a fragment ion can perhaps be rationalised by acknowledging the range of reagent ions formed under these conditions, many of which can give rise to ionisation with concomitant dissociation.^10^ The ions formed are summarised in Table 2. The use of N_2_ humidified with water vapour also generates a range of oxygenated species for hydrocarbons originating from base oil. These ions are noted in greater abundance in the total ion current chromatogram trace later than the ions generated for the antioxidants, shown by Figure S3. Ions generated for antioxidants and base oil during the course of the analysis are given by the spectra in Figure 1. The findings that both antioxidants and base oil hydrocarbons can be analysed rapidly under these conditions in the same run are of particular interest; such concomitant analysis of non‐ and semi‐polar compounds cannot be demonstrated with a more commonplace ion source such as ESI.

**TABLE 2:** Ions observed across all analytical conditions in both positive and negative ion mode

Additive	Representative structure	Composition range	Ion type	Theoretical mass (Da) of select ions	Conditions (and MS polarity)
Aminic antioxidant	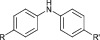	R + R' = C_4_H_10_ to R + R' = C_16_H_34_	[M + H]^+^	226.1590	All conditions (+)
				282.2216	
				338.2842	
				394.3468	
Phenolic antioxidant	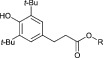	R = C_7_H_15_ to R = C_9_H_19_	[M − CH_3_]^+^	375.2894	Dry N_2_ (+)
			[M]^•+^	390.3128	N_2_ with water vapour (+)
			[M − H]^+^	389.3050	
			[M − C_4_H_9_]^+^	335.2581	
			[M + NH_4_]^+^	408.3472	
			[M − H]^−^	389.3061	All conditions (−)
			[M − 3H]^−^	387.2905	
Base oil	Hydrocarbon isomers	C_9_H_20_ marker alkane	[M − 5H + 2O]^+^	155.1067	N_2_ with water vapour (+)
		C_7_H_16_ marker alkane	[M − 7H + 5O]^−^	173.0443	N_2_ with water or alcohol vapour (−)
Dispersant	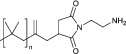	n = 7–9^a^	[M + H]^+^	589.5667	N_2_ with acetonitrile vapour (+), *concentrated sample*
ZDDP ligand	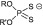	R + R' = C_6_H_14_ to R + R' = C_12_H_26_	[M]^−^	213.0178	All conditions (−)
				255.0648	
				297.1117	

In all instances, ions were detected with mass differences of less than 2 mmu from theoretical masses.

^a^ Spectra for dispersant ions are shown in Figure S4 (supporting information).

Replacing water in the bubbler with the vapour‐generating solvents methanol, isopropanol or acetonitrile saw aminic antioxidants remain the most readily observed FFO component, yielding [M + H]^+^ ions. This is consistent with the literature detailing protonation of amines in DBDI without the water‐mediated generation of hydroxonium reagent ions.^16^ Notably, no ions related to phenolic antioxidant are observed when organic solvents are used to generate a vapour.

Tentative identification of protonated alkyl succinimide dispersant additives from diesel fuel samples has been previously reported using direct analysis in real time (DART)‐MS under different analytical conditions.^17^ The observation of protonated alkyl succinimide dispersant ions in an FFO using this experimental set‐up is also possible when using acetonitrile vapours to ionise an FFO sample of concentration eight times greater than used for typical analyses, albeit yielding ions with low intensity. These ions are shown in Figure S4. The range of ions of interest observed in positive ion mode for FFO components across all analytical conditions is reported in Table 2. The lack of ions observed for the viscosity modifier and antifoam additives may possibly be attributed to the high molecular weight of these materials or concentration of these analytes within the formulation.

### 3.2 Negative ion mode DBDI‐MS

When operating plasma‐based sources using air as a discharge gas, several species have been identified that could contribute to the formation of reagent ions for negative ion mode DBDI, such as oxygen‐based radical anions, in addition to NO_3_^−^ and NO_2_^−^.^7^, ^18^ Eventual ionisation of analytes leads to a range of common ion types, such as radical and deprotonated anions, products of ion attachment and oxygenation as well as fragmentation in some instances.^7^, ^19^, ^20^

Negative ion mode analyses were conducted that complement the conditions used for positive ion mode analyses reported above, leading to the observation of phenolic antioxidant and small ZDDP ligand ions being observed under all conditions. The generation of small ZDDP ligand ions in all cases may be rationalised as the ZDDP complex releasing the constituent anionic thiophosphate ligands.^4^ Under all conditions, the phenolic antioxidant is not only detected as a simple [M − H]^−^ deprotonated species, but also as ions of the type [M − 3H]^−^, seemingly having undergone additional dehydrogenation. Loss of the relatively acidic phenolic hydrogen as a proton to form a [M − H]^−^ ion is a facile process and would occur readily by reaction with anionic reagent ions; however, the concomitant dehydrogenation observed is less easily rationalised, although such a loss would enable greater stabilisation of a charge via resonance. An example spectrum of an FFO analysis using the conditions discussed above can be seen in Figure S5.

**FIGURE 2:**
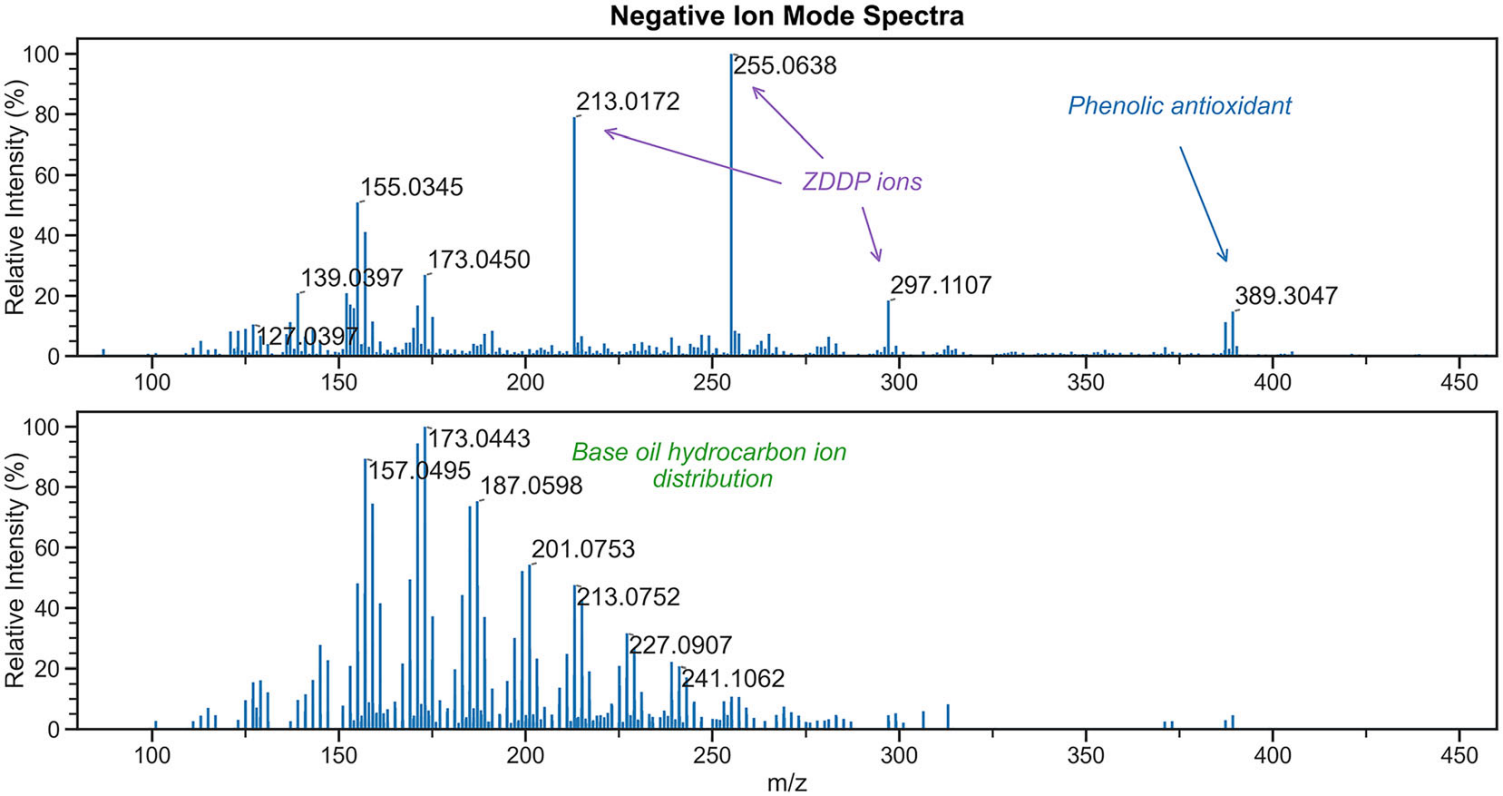
Mass spectra of FFO generated in negative ion mode. Initial ionisation of additive components at 0.07 min (top) followed by subsequent ionisation of hydrocarbon base oil at 0.30 min in the same analysis (bottom), when using N_2_ humidified with water vapour as a discharge gas [Color figure can be viewed at wileyonlinelibrary.com]

In addition, when water or an alcohol solvent vapour is presented to the source through the bubbler, a hydrocarbon distribution was also identified corresponding to the formation of highly oxygenated species from base oil, shown in Figure 2. This is in contrast with analyses in positive ion mode, where a hydrocarbon distribution is only observed when using water vapour; the ions generated in positive ion mode typically contain a lower oxygen content. The observation of O_2_^−^ attachment ions has been reported for saturated alkanes analysed using DART in negative ion mode; however, using the set‐up described for the present study, ions containing three or more oxygen atoms are predominately observed.^21^ The range of ions formed in negative ion mode is summarised in Table 2, with spectra given in Figures S5–S7. Notably, no detergent ions were detected under any conditions in negative ion mode, despite these analytes being anionic in solution.

### 3.3 Petroleomic visualisations

**FIGURE 3:**
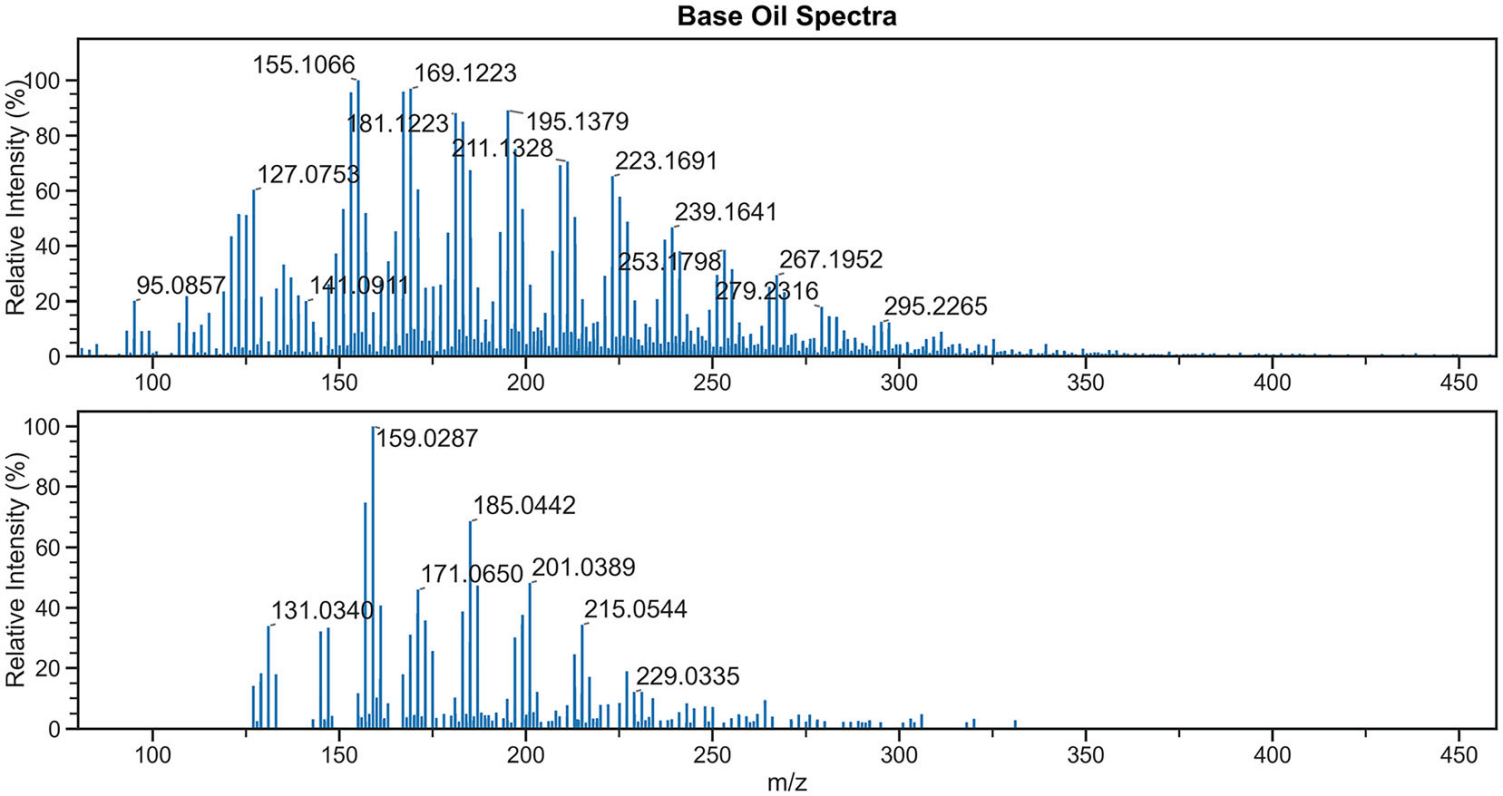
Mass spectra of FFO base oil in positive (top) and negative (bottom) ion mode at 0.40 min of each analysis. Background subtraction has been performed. Nitrogen humidified with water vapour was used as the discharge gas [Color figure can be viewed at wileyonlinelibrary.com]

Comparison of ions generated in positive and negative ion modes for hydrocarbon base oil when using N_2_ humidified with water vapour as the discharge gas shows two distinctly different distributions, shown in Figure 3. All following analyses focusing on analysing the hydrocarbon base oil used a SPME heater temperature of 320°C. This temperature was used to ensure maximum transfer of base oil hydrocarbons into the gas phase for ionisation within the source; however, it is accepted that at near‐atmospheric pressure this temperature is unlikely to lead to vaporisation of the entire base oil hydrocarbon range.

In order to comprehensively profile differences between ions generated in both polarities, visualisation of the datasets was conducted using common petroleomic techniques, including KMD and van Krevelen plots.^22^, ^23^ KMD plots make use of the exact mass of a repeat unit, typically CH_2_, to generate a Kendrick mass scale. Using this new mass scale, ions generated in complex mixtures can be sorted by their class type into corresponding homologous series and be subsequently visualised. This treatment can allow rapid identification of related ions from relatively complex spectra. Van Krevelen plots visualise the correlation between the hydrogen–carbon and oxygen–carbon ratios of ions, and can help profile information on ion compositions, such as extent of oxygenation and desaturation within complex mixtures.

Typically, an aim of petroleomic analysis is assignment and profiling of elemental compositions where mixtures can contain a range of heteroatoms and classes; however, in this case this type of analysis is applied to the assessment of classes of ions formed upon ionisation using the DBDI source. It is known that the base oil components should be comprised of mostly carbon and hydrogen, and be predominantly saturated, due to its classification as an American Petroleum Institute (API) Group III base oil. Therefore, the main factor influencing the range of ions and classes generated will be reactions undergone by the compounds within and subsequent to the DBDI source.

**FIGURE 4:**
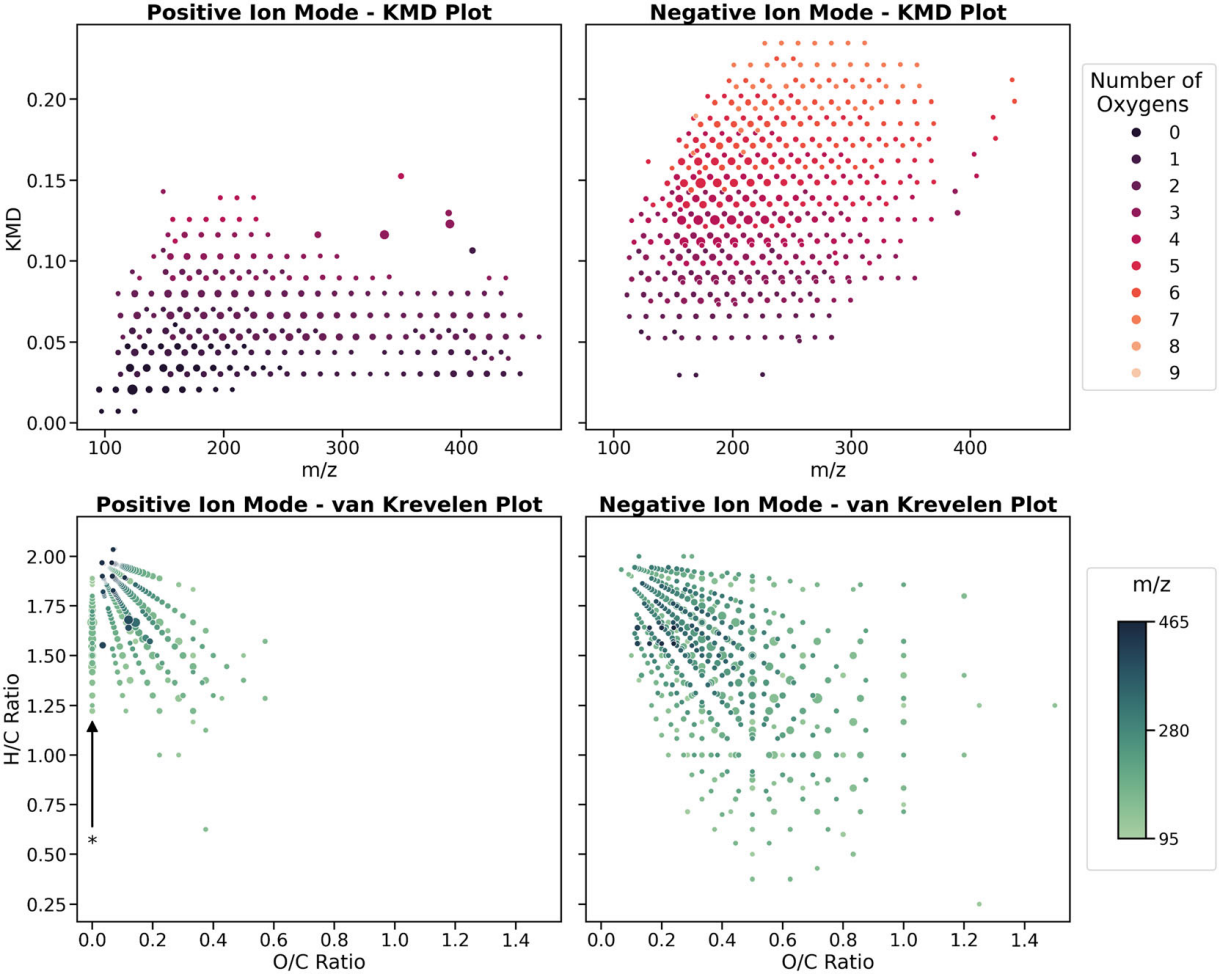
KMD plots (top row) for ions generated from hydrocarbon base oil in positive and negative ion modes. Van Krevelen plots (bottom row) for ions from the same dataset. A larger size of data point indicates a higher relative intensity. All analyses were conducted using N_2_ humidified with water vapour as a discharge gas. The series marked with an asterisk in the positive ion mode van Krevelen plot is discussed in more detail later in the text [Color figure can be viewed at wileyonlinelibrary.com]

To prepare the data for analysis, spectra were averaged between 0.2 and 0.4 min, elemental compositions were assigned to ions between 80 and 500 *m*/*z* above a threshold of 2% relative abundance, and the subsequent mass list exported. Assessing the base oil hydrocarbon ions formed using both KMD and van Krevelen plots given by Figure 4, both indicate an increased oxygen content and decreased saturation of ions formed in negative ion mode with respect to ions formed in positive ion mode. In the KMD plots, this is given by higher overall KMD values for ions generated in negative ion mode. Increased oxygen content of ions has been shown in the literature to yield higher KMD values, and is more visible if ions in the KMD plot are colour‐coded by their oxygen content.^24^.

Furthermore, van Krevelen plots can be useful for mapping the composition range of ions formed in complex mixtures. By plotting the hydrogen–carbon ratio against the oxygen–carbon ratio, it is possible to identify relationships between the degree of unsaturation of the ions and their oxygen content. A general negative correlation between these two variables can be observed, indicating that the degree of saturation decreases with increasing oxygen content. This trend is much more diffuse in negative ion mode, extending to much higher O/C ratios. Also of interest is the distribution of ions in positive ion mode where O/C = 0, indicating the formation of cations without oxygen, marked in Figure 4 with an asterisk. This is further discussed when analysing the dataset using oxygen‐content bar plots below.

In a separate investigation, N_2_ containing methanol or isopropanol vapours used as a discharge gas was also found to generate anionic species for hydrocarbon base oil and were compared to the range of ions generated when using N_2_ humidified with water vapour. Visualisation of these datasets using van Krevelen plots shows many similar motifs to those obtained from analyses using water, given by the bottom right plot in Figure 4. Additional van Krevelen, KMD and bar plots are available in Figures S8 and S9 for this study.

**FIGURE 5:**
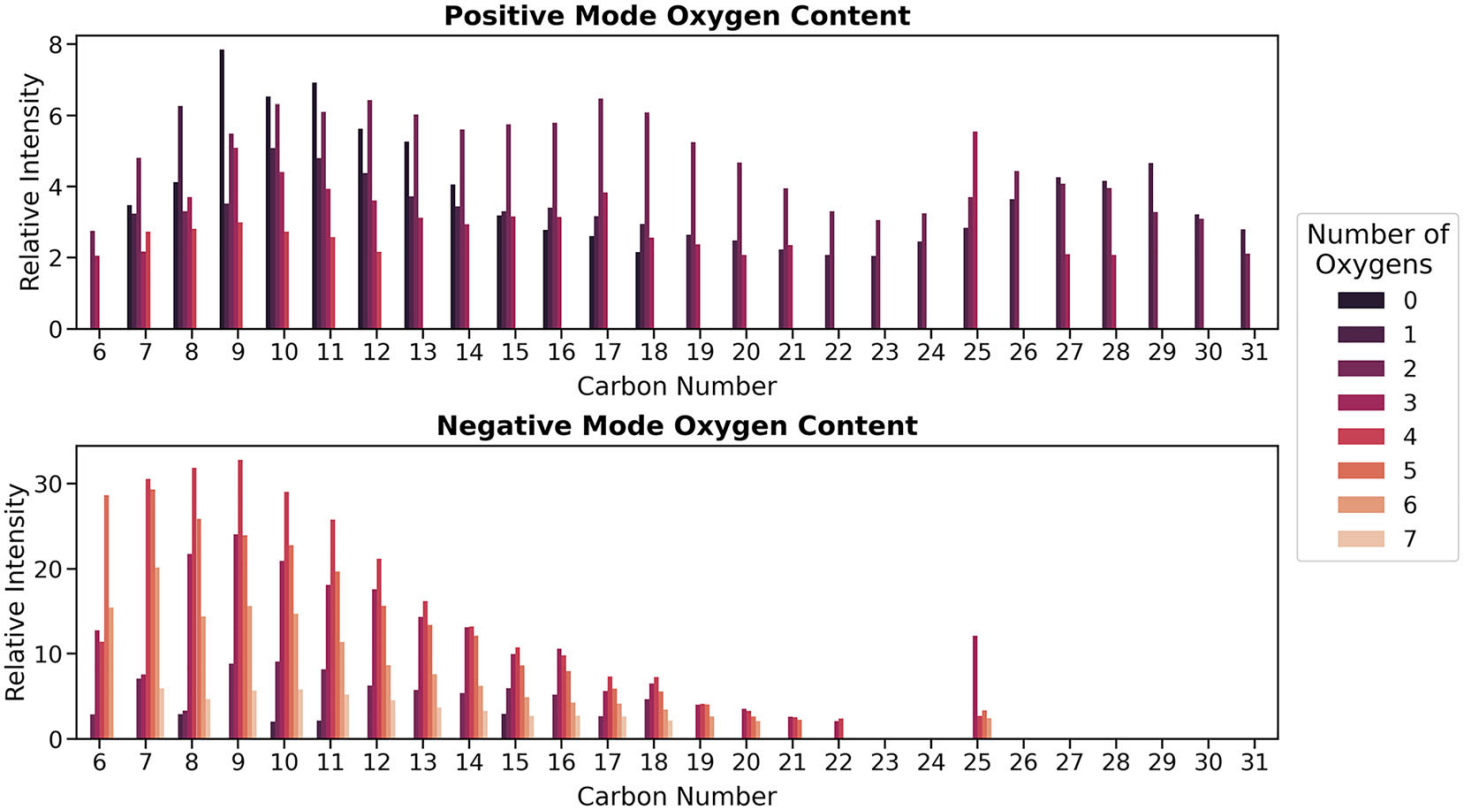
Oxygen content distribution for FFO hydrocarbon ions generated using N_2_ humidified with water vapour as a discharge gas in both positive and negative ion modes. Discontinuous data at 25 carbons in both polarities arises from ionisation of residual phenolic antioxidant [Color figure can be viewed at wileyonlinelibrary.com]

Visualising finer details in trends of ion composition over a hydrocarbon distribution can be easier when carbon number is chosen as a plotting variable. The preparation of a bar plot allows additional variables such as double bond equivalent values or oxygen number to be reported, which can readily provide information on ion composition trends. Figure 5 shows the distribution of oxygen content across carbon number for hydrocarbon ions in both polarities and confirms increased incorporation of oxygen in negative ion mode compared to positive ion mode. In addition, the data show that ions observed in negative ion mode typically contain more than three oxygen atoms, whereas the upper limit for number of oxygens contained within ions observed in positive ion mode is four. This stark contrast is of interest and suggests the formation of significantly different ions between the two polarities. This is further substantiated by the lack of ions in negative ion mode with greater than 22 carbons, suggesting that the ionisation pathway in the negative ion mode leads to higher incidences of fragmentation, particularly of larger ions. Discontinuous data at 25 carbons in both polarities arises from ionisation of residual phenolic antioxidant. Poor representation of base oil hydrocarbons with a chain length above approximately 30 carbons using this technique, compared to more typical FI‐MS analyses, is attributed to a limitation of not performing ionisation under near‐vacuum conditions where transfer of sparingly volatile hydrocarbons into the gas phase is much more pronounced. An FI spectrum for this base oil is given in Figure S10, and evidences a hydrocarbon range of chain length in excess of 30 carbons.

**FIGURE 6:**
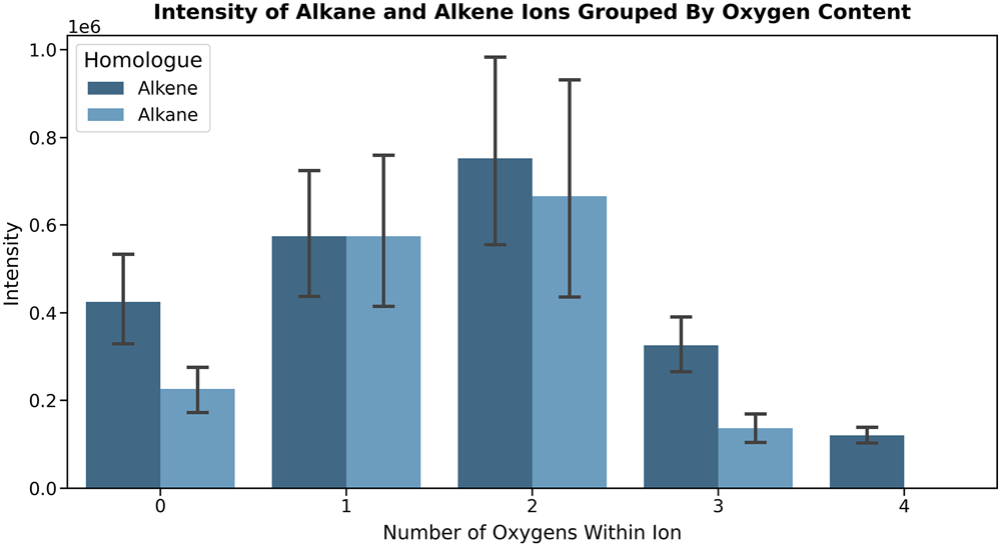
Ratio of ions formed for the selected alkanes and alkenes, grouped by oxygen content. The data indicate that similar ions are formed from both alkenes and alkanes; however, the increased formation of zero‐ and three‐oxygen species from alkenes compared to alkanes is statistically significant and suggests the alkene moiety promotes the formation of these species. Error bars show the 95% confidence intervals from five repeat measurements [Color figure can be viewed at wileyonlinelibrary.com]

It was considered that the trend at O/C = 0 in the positive ion mode van Krevelen plot (Figure 4) marked with an asterisk may arise from ionisation of a small fraction of alkene components within the FFO base oil, where a putative initial hydride removal would be stabilised via allylic resonance and provide a pathway to forming stable ions without incorporation of oxygen. By classification, API Group III base oils, such as that used in the present work, may contain less than or equal to 10% unsaturated hydrocarbon content, suggesting that the presence of some alkenes is likely.^25^ Hydride abstraction has been posited as the initial step in the ionisation of alkanes using DBDI, where the formed ions undergo additional reactions in many cases.^15^ To investigate the hypothesis of stabilised hydride abstraction in alkene ionisation, two linear alkenes, namely 1‐decene and 1‐dodecene, in addition to two linear alkanes, namely *n*‐decane and *n*‐dodecane, were analysed. Visualisation of the ratios of ions generated allows the propensity of ion formation to be compared (Figure 6). To export the mass list, elemental compositions were assigned within Qual Browser to ions over a mass window of 80–240 *m*/*z* and above a 2% relative intensity threshold. Inspection of ions below 2% relative intensity was conducted manually and in some instances confirmed the presence of some very minor products of alkane or alkene ionisation; however, these are poorly distinguished from the noise and so the threshold was maintained at 2% relative intensity for exporting mass lists. Data from five repeats were exported and used to plot 95% confidence intervals. Ions containing nitrogen were excluded from the data visualisation.

Comparing the ions formed for all C_10_ and C_12_ compounds in Figure 6, we see that the increased formation of zero‐oxygen species from alkenes is statistically significant when compared to the same classes generated from alkanes. This is in contrast with one‐ and two‐oxygen species, which appear to yield similar ion intensities irrespective of being formed from either alkanes or alkenes. We also see that alkenes produce a markedly larger intensity of three‐oxygen species compared to alkanes, suggesting that the presence of an alkene moiety promotes formation of zero‐ and three‐oxygen species compared to alkane counterparts.

## 4 CONCLUSIONS

The analysis of an FFO using active capillary DBDI‐MS has been developed and the influence of solvent vapours doped into the discharge gas as ionisation modifiers investigated. Through selection of an appropriate solvent, or lack of solvent altogether, various engine oil components can be selectively profiled, leading to targeted analyses. In positive ion mode, some additives are observed through the formation of simple [M + H]^+^ ions. Notable exceptions to this case are the range of ions generated when water vapour is present within the discharge gas for the phenolic antioxidant and base oil hydrocarbons. In a similar fashion, complementary analyses in negative ion mode led to the observation of more complex ions for both phenolic antioxidant and base oil constituents but yielded simple anionic species for ZDDP ligands.

Visualisation of the ions generated for the base oil component of the FFO has been conducted using commonplace petroleomic techniques, detailing how different analytical conditions alter the ionisation of a complex mixture of hydrocarbons. In all instances, a wide range of ions were observed for base oil hydrocarbons resulting from differing extents of oxygenation and dehydrogenation. Furthermore, the study of ions formed from selected linear alkanes and alkenes suggests that the presence of an alkene moiety can promote the formation of zero‐ and three‐oxygen species when compared to the corresponding alkanes in positive ion mode.

In addition to these findings, the generation of species in negative ion mode DBDI‐MS for hydrocarbons not yet disclosed elsewhere in the literature has been reported. Analyses using this technique are rapid, taking less than 1 min to generate a good overview of composition, and could prove to be of use in analysing a range of different real‐world engine oil formulations. Moreover, coverage of both selected additives and the base oil component of the formulation is novel and, to the knowledge of the authors, not readily accessible through other ion sources.

### PEER REVIEW

The peer review history for this article is available at https://publons.com/publon/10.1002/rcm.9320.

## Supporting information


**Figure S1.** DBDI‐MS setup
**Figure S2.** Mass spectrum of phenolic antioxidant ions generated using humidified and dry nitrogen as a discharge gas
**Figure S3.** Extracted Ion Chromatogram for a typical analysis
**Figure S4.** Mass spectrum of dispersant ions
**Figure S5.** Mass spectrum of ions typically observed in negative ion mode
**Figure S6.** Hydrocarbon ions generated in negative ion mode
**Figure S7.** Complex ZDDP ions formed in negative ion mode
**Figure S8.** KMD plots for hydrocarbon base oil using different solvents in negative ion mode
**Figure S9.** Oxygen content of hydrocarbon ions formed in negative ion mode
**Figure S10.** FIMS Spectrum of Base Oil

## Data Availability

The data that supports the findings of this study are available in the supplementary material of this article

## References

[1] Kreisberger G , Klampfl CW , Buchberger WW . Determination of antioxidants and corresponding degradation products in fresh and used engine oils. Energy Fuel. 2016;30(9):7638‐7645. doi:10.1021/acs.energyfuels.6b01435

[2] Ahmed NS , Abdel‐Hameed HS , El‐Kafrawy AF , Nassar AM . Synthesis and evaluation of ashless detergent/dispersant additives for lubricating engine oil. Ind Lubr Tribol. 2015;67(6):622‐629. doi:10.1108/ILT-05-2015-0065

[3] Barnes AM , Bartle KD , Thibon VRA . A review of zinc dialkyldithiophosphates (ZDDPs): Characterisation and role in the lubricating oil. Tribol Int. 2001;34:389‐395. www.elsevier.com/locate/triboint. Accessed August 2, 2017

[4] Spikes H . The history and mechanisms of ZDDP. Tribol Lett. 2004;17(3):469‐489. doi:10.1023/B:TRIL.0000044495.26882.b5

[5] Kassler A , Pittenauer E , Dörr N , Allmaier G . Ultrahigh‐performance liquid chromatography/electrospray ionization linear ion trap Orbitrap mass spectrometry of antioxidants (amines and phenols) applied in lubricant engineering. Rapid Commun Mass Spectrom. 2014;28(1):63‐76. doi:10.1002/rcm.6756 24285391

[6] Dörr N , Agocs A , Besser C , Ristić A , Frauscher M . Engine oils in the field: A comprehensive chemical assessment of engine oil degradation in a passenger car. Tribol Lett. 2019;67(3):68. doi:10.1007/s11249-019-1182-7

[7] Gyr L , Wolf JC , Franzke J , Zenobi R . Mechanistic understanding leads to increased ionization efficiency and selectivity in dielectric barrier discharge ionization mass spectrometry: A case study with perfluorinated compounds. Anal Chem. 2018;90(4):2725‐2731. doi:10.1021/acs.analchem.7b04711 29356499

[8] Huba AK , Mirabelli MF , Zenobi R . Understanding and optimizing the ionization of polycyclic aromatic hydrocarbons in dielectric barrier discharge sources. Anal Chem. 2019;91(16):10694‐10701. doi:10.1021/acs.analchem.9b02044 31337215

[9] Martínez‐Jarquín S , Winkler R . Low‐temperature plasma (LTP) jets for mass spectrometry (MS): Ion processes, instrumental set‐ups, and application examples. TrAC Trends Anal Chem. 2017;89:133‐145. doi:10.1016/j.trac.2017.01.013

[10] Gyr L , Klute FD , Franzke J , Zenobi R . Characterization of a nitrogen‐based dielectric barrier discharge ionization source for mass spectrometry reveals factors important for soft ionization. Anal Chem. 2019;91(10):6865‐6871. doi:10.1021/acs.analchem.9b01132 31035763

[11] Venter AR , Douglass KA , Shelley JT , Hasman G , Honarvar E . Mechanisms of real‐time, proximal sample processing during ambient ionization mass spectrometry. Anal Chem. 2014;86(1):233‐249. doi:10.1021/ac4038569 24308499

[12] Zuppa Neto TD , Avval TG , Morais PA , et al. Direct dielectric barrier discharge ionization promotes rapid and simple lubricant oil fingerprinting. J Am Soc Mass Spectrom. 2020;31(7):1525‐1535. doi:10.1021/jasms.0c00071 32453588

[13] Mirabelli MF , Wolf JC , Zenobi R . Direct coupling of solid‐phase microextraction with mass spectrometry: Sub‐pg/g sensitivity achieved using a dielectric barrier discharge ionization source. Anal Chem. 2016;88(14):7252‐7258. doi:10.1021/acs.analchem.6b01507 27332082

[14] Mirabelli MF , Wolf JC , Zenobi R . Atmospheric pressure soft ionization for gas chromatography with dielectric barrier discharge ionization‐mass spectrometry (GC‐DBDI‐MS). Analyst. 2017;142(11):1909‐1915. doi:10.1039/c7an00245a 28443843

[15] Weber M , Wolf JC , Haisch C . Gas chromatography‐atmospheric pressure inlet‐mass spectrometer utilizing plasma‐based soft ionization for the analysis of saturated, aliphatic hydrocarbons. J Am Soc Mass Spectrom. 2021;32(7):1707‐1715. doi:10.1021/jasms.0c00476 34170138

[16] Wolf JC , Gyr L , Mirabelli MF , Schaer M , Siegenthaler P , Zenobi R . A radical‐mediated pathway for the formation of [M + H]^+^ in dielectric barrier discharge ionization. J Am Soc Mass Spectrom. 2016;27(9):1468‐1475. doi:10.1007/s13361-016-1420-2 27380388

[17] Barnett I , Zhang M . Discrimination of brands of gasoline by using DART‐MS and chemometrics. Forensic Chem. 2018;10:58‐66. doi:10.1016/j.forc.2018.07.003

[18] Donò A , Paradisi C , Scorrano G . Abatement of volatile organic compounds by corona discharge. A study of the reactivity of trichloroethylene under atmospheric pressure ionization conditions. Rapid Commun Mass Spectrom. 1997;11(15):1687‐1694. doi:10.1002/(SICI)1097-0231(19971015)11:15<1687::AID-RCM33>3.0.CO;2-Y

[19] Na N , Zhang C , Zhao M , et al. Direct detection of explosives on solid surfaces by mass spectrometry with an ambient ion source based on dielectric barrier discharge. J Mass Spectrom. 2007;42(8):1079‐1085. doi:10.1002/jms.1243 17618527

[20] Hagenhoff S , Korf A , Markgraf U , et al. Screening of semifluorinated *n*‐alkanes by gas chromatography coupled to dielectric barrier discharge ionization mass spectrometry. Rapid Commun Mass Spectrom. 2018;32(13):1092‐1098. doi:10.1002/rcm.8139 29660193

[21] Cody RB , Dane AJ . Dopant‐assisted direct analysis in real time mass spectrometry with argon gas. Rapid Commun Mass Spectrom. 2016;30(10):1181‐1189. doi:10.1002/rcm.7552 28328019

[22] Catalina D , Lozano P , Thomas MJ , Jones HE , Barrow MP . Petroleomics: Tools, challenges, and developments. Annu Rev Anal Chem. 2020;13(1):405‐430. doi:10.1146/annurev-anchem-091619 32197051

[23] Wu Z , Rodgers RP , Marshall AG . Two‐ and three‐dimensional van Krevelen diagrams: A graphical analysis complementary to the Kendrick mass plot for sorting elemental compositions of complex organic mixtures based on ultrahigh‐resolution broadband Fourier transform ion cyclotron resonance. Anal Chem. 2004;76(9):2511‐2516. doi:10.1021/ac0355449 15117191

[24] Sleno L . The use of mass defect in modern mass spectrometry. J Mass Spectrom. 2012;47(2):226‐236. doi:10.1002/jms.2953 22359333

[25] API Base Oil Interchangeability Guidelines for Passenger Car Motor Oils and Diesel Engine Oils E.1 General. 2015. http://www.api.org/~/media/files/certification/engine-oil-diesel/publications/anne-rev-03-25-15.pdf. Accessed October 21, 2021.

