# Supplementary material for: Detailed chemical analysis of a fully formulated oil using dielectric barrier discharge ionisation–mass spectrometry
Source: Rapid Commun Mass Spectrom. 2022 May 16;36(14):e9320. doi: 10.1002/rcm.9320 (PMC9286547; doi:10.1002/rcm.9320)
Supplement: Supplementary file 1 — Figure S1. DBDI‐MS setup Figure S2. Mass spectrum of phenolic antioxidant ions generated using humidified and dry nitrogen as a discharge gas Figure S3. Extracted Ion Chromatogram for a typical analysis Figure S4. Mass spectrum of dispersant ions Figure S5. Mass spectrum of ions typically observed in negative ion mode Figure S6. Hydrocarbon ions generated in negative ion mode Figure S7. Complex ZDDP ions formed in negative ion mode Figure S8. KMD plots for hydrocarbon base oil using different solvents in negative ion mode Figure S9. Oxygen content of hydrocarbon ions formed in negative ion mode Figure S10. FIMS Spectrum of Base Oil [file RCM-36-0-s001.docx]

**Supplementary Information - Detailed chemical analysis of a fully formulated oil using dielectric barrier discharge ionisation-mass spectrometry**

Contents

[Figure S1 – DBDI-MS setup 2](#_Toc88812638)

[Figure S2 – Mass spectrum of phenolic antioxidant ions generated using humidified and dry nitrogen as a discharge gas 3](#_Toc88812639)

[Figure S3 – Extracted Ion Chromatogram for a typical analysis 3](#_Toc88812640)

[Figure S4 – Mass spectrum of dispersant ions 4](#_Toc88812641)

[Figure S5 – Mass spectrum of ions typically observed in negative ion mode 4](#_Toc88812642)

[Figure S6 – Hydrocarbon ions generated in negative ion mode 5](#_Toc88812643)

[Figure S7 – Complex ZDDP ions formed in negative ion mode 5](#_Toc88812644)

[Figure S8 – KMD plots for hydrocarbon base oil using different solvents in negative ion mode 6](#_Toc88812645)

[Figure S9 – Oxygen content of hydrocarbon ions formed in negative ion mode 6](#_Toc88812646)

[Figure S10 – FIMS Spectrum of Base Oil 7](#_Toc88812647)

# Figure S1 – DBDI-MS setup


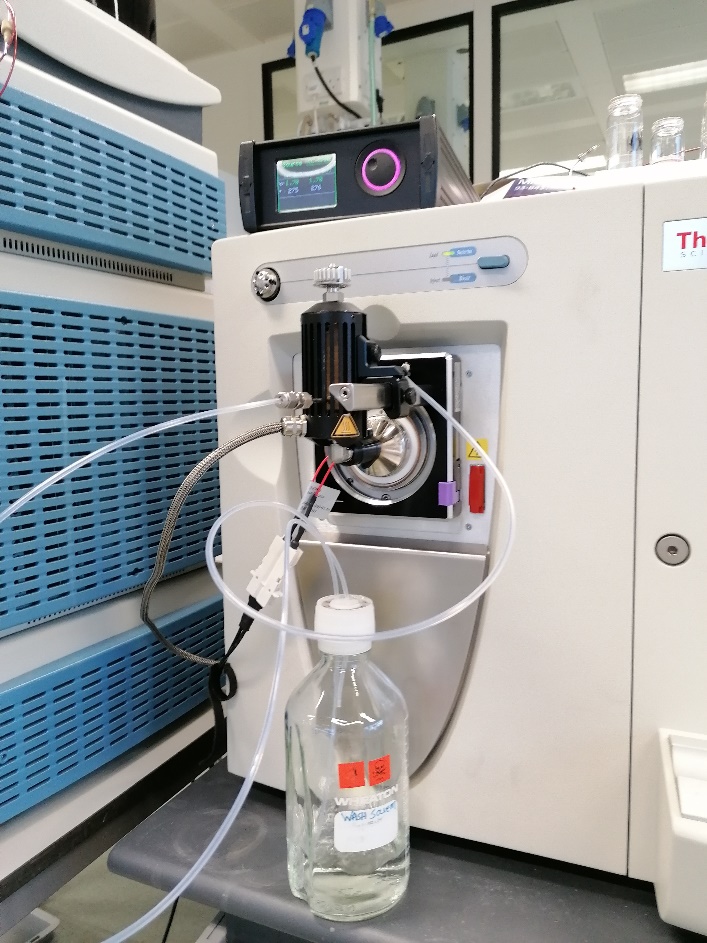


*Figure S1: experimental setup of SICRIT source and heating unit with bubbler attached.*

# Figure S2 – Mass spectrum of phenolic antioxidant ions generated using humidified and dry nitrogen as a discharge gas

[M+NH_4_]^+^

[M-CH_3_]^+^

[M]^·+^

[M-C_4_H_9_]^+^

*Figure S2: Mass spectra generated when analysing an FFO using nitrogen humidified with water vapour (top) as a discharge gas, and using dry nitrogen (bottom) as a discharge gas. Ion assignments are for phenolic antioxidant analyte.*

# Figure S3 – Extracted Ion Chromatogram for a typical analysis


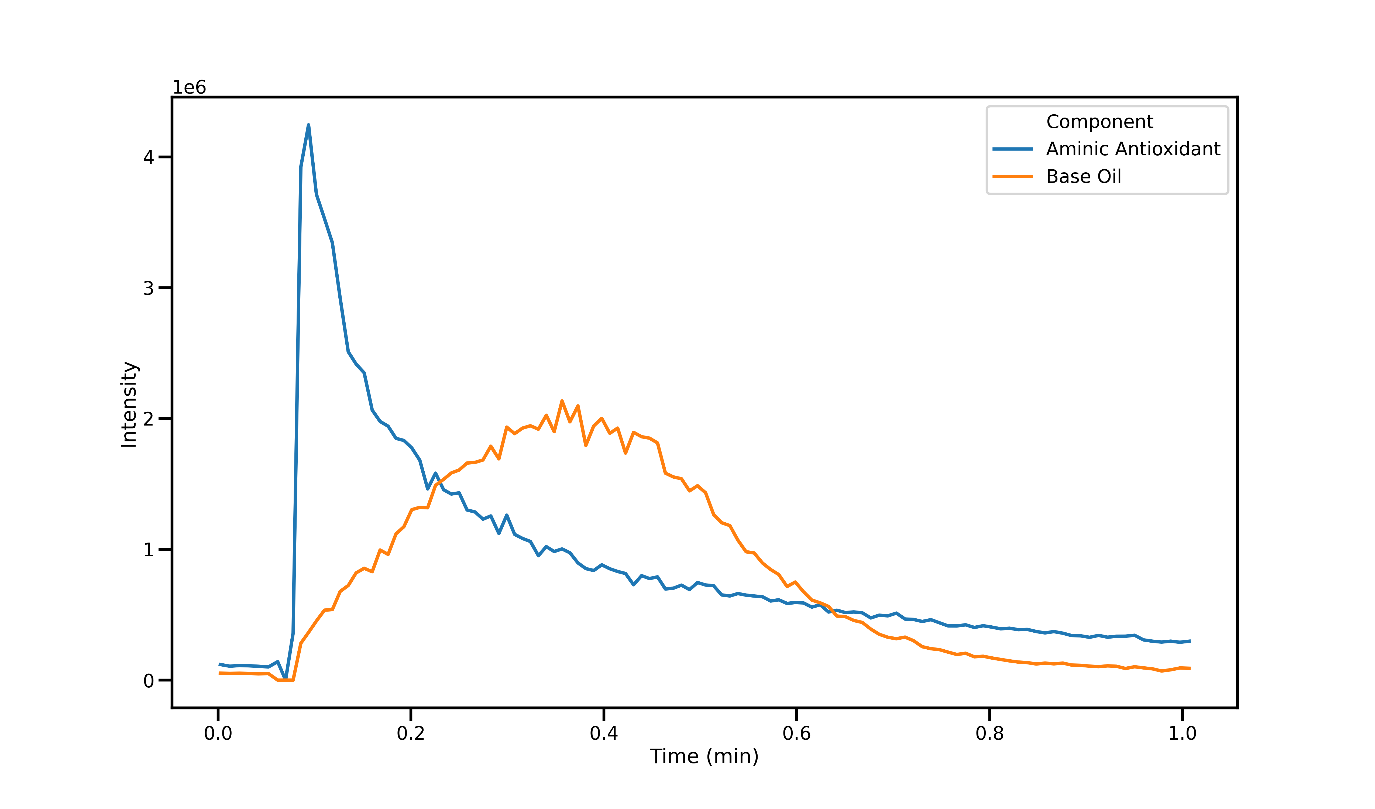


*Figure S3: Ion intensities over time for aminic antioxidant (blue) and base oil (orange) when using humidified nitrogen as a discharge gas.*

# Figure S4 – Mass spectrum of dispersant ions

[M+H]^+^

[M+H]^+^

[M+H]^+^

*Figure S4: mass spectrum detailing ionisation of dispersants. These are assigned as [M+H]^+^ ions at 533, 589, and 645 m/z.*

# Figure S5 – Mass spectrum of ions typically observed in negative ion mode

[M-3H]^-^ Phenolic Antioxidant

[C_6_H_14_O_2_PS_2_]^-^

ZDDP

[C_12_H_26_O_2_PS_2_]^-^

ZDDP

[C_9_H_20_O_2_PS_2_]^-^

ZDDP

*Figure S5: Range of additive ions typically observed in negative ion mode under all conditions. Spectrum acquired at 0.06 minutes of analysis using humidified nitrogen as a discharge gas.*

# Figure S6 – Hydrocarbon ions generated in negative ion mode

*Figure S6: A magnified region of hydrocarbon ions produced when using humidified nitrogen as a discharge gas in negative ion mode, detailing variety of ions generated.*

# Figure S7 – Complex ZDDP ions formed in negative ion mode

*Figure S7: Range of ZDDP ions formed when using IPA as a discharge gas modifier, identified by a characteristic isotope distribution for zinc.*

# Figure S8 – KMD plots for hydrocarbon base oil using different solvents in negative ion mode


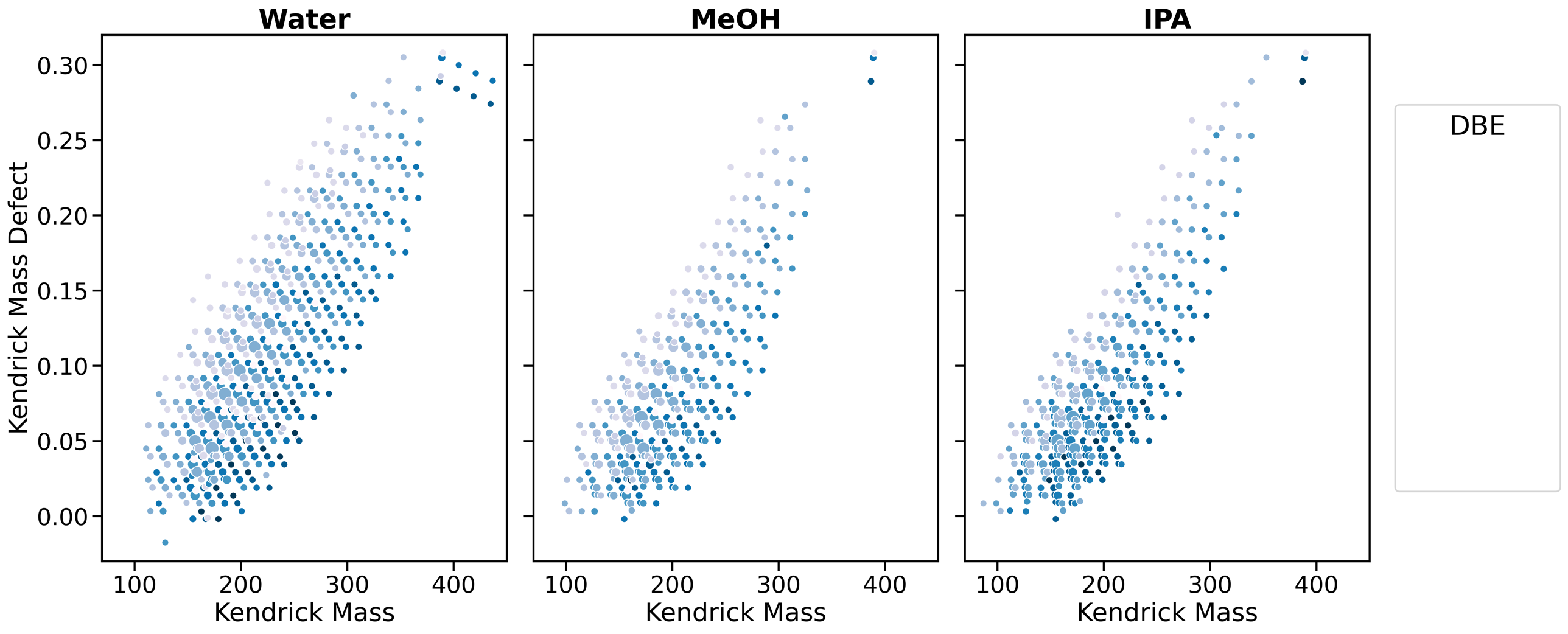

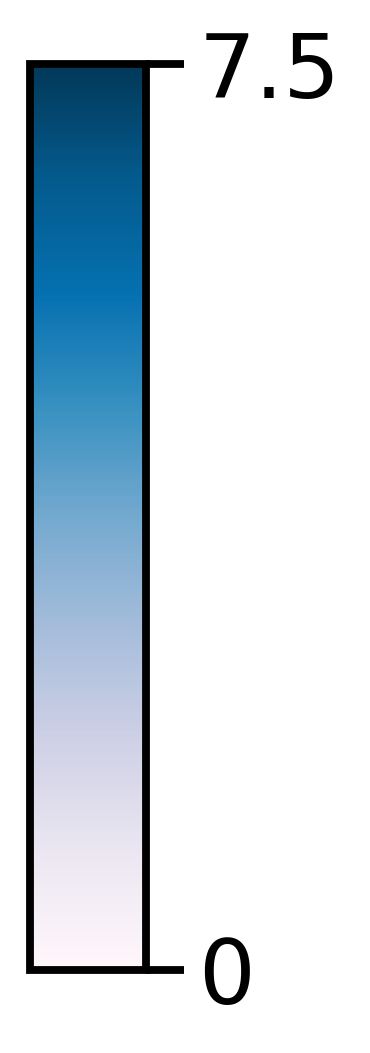


*Figure S8: KMD plots for hydrocarbon base oil ions generated when using different solvents as a discharge gas modifier.*

# Figure S9 – Oxygen content of hydrocarbon ions formed in negative ion mode


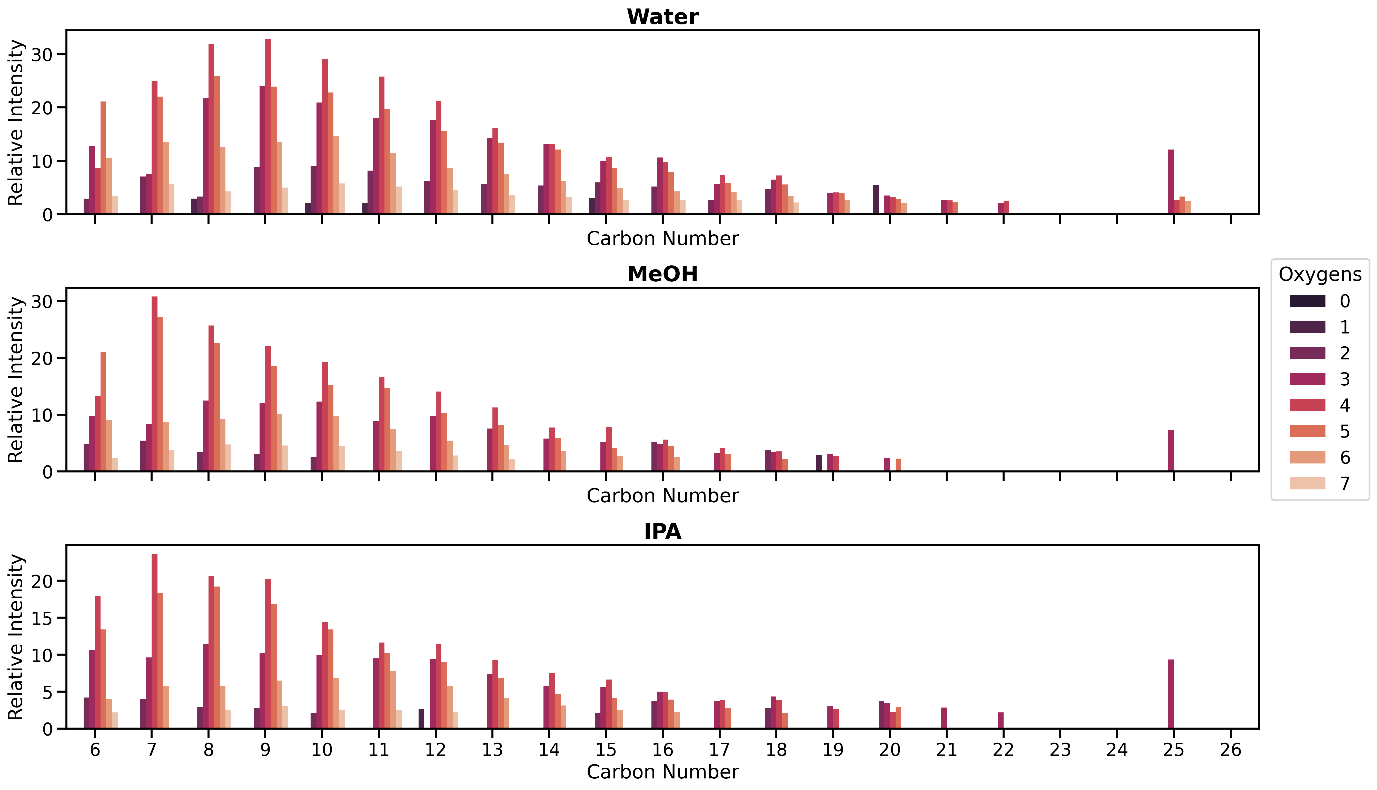


*Figure S9: bar plots for distribution of oxygen content within ions generated from hydrocarbon base oil using different solvents to modify the discharge gas in negative ion mode.*

# Figure S10 – FIMS Spectrum of Base Oil


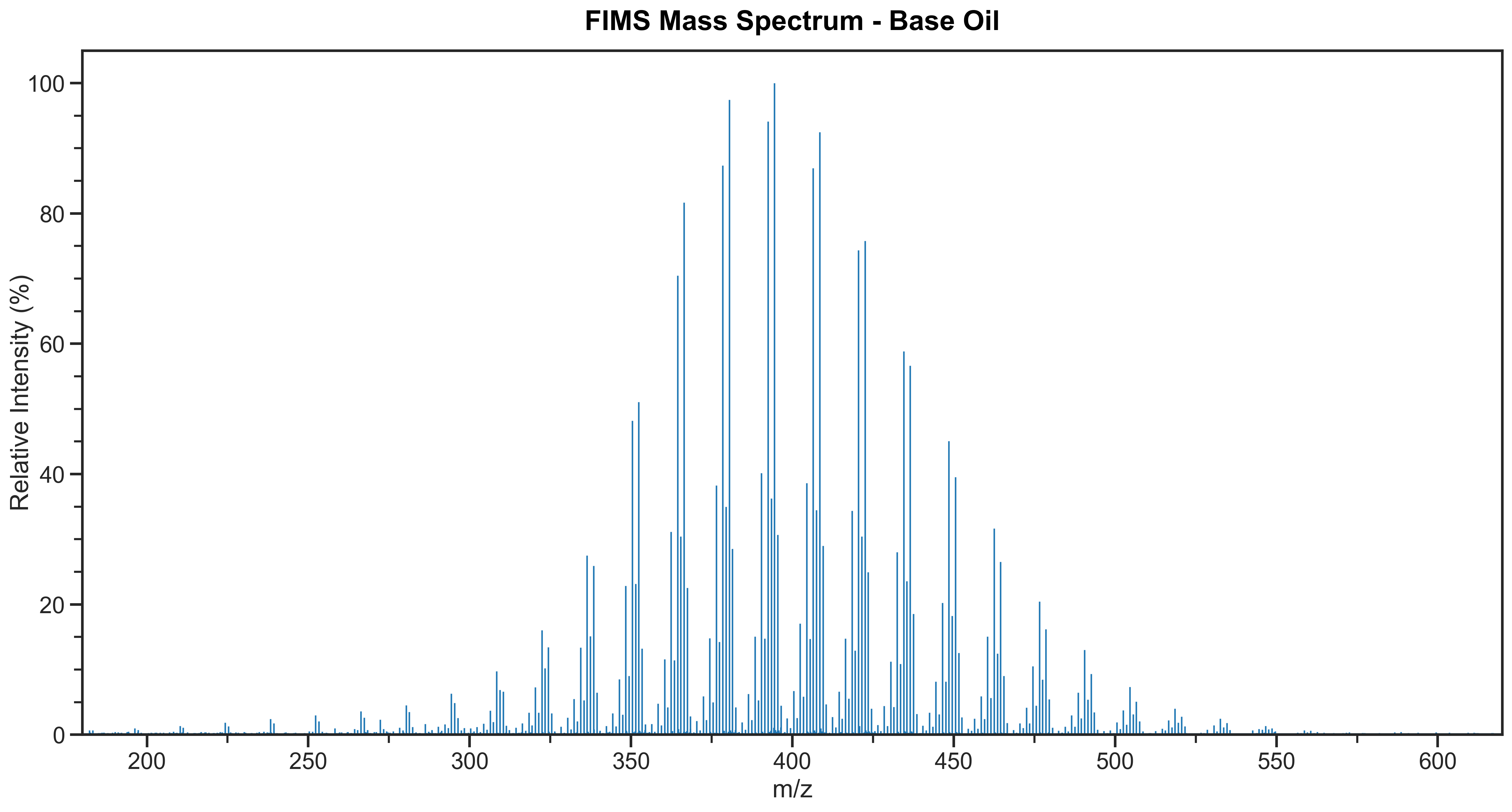


*Figure S10: FIMS positive ion mode spectrum of base oil*
